# Isolation of exosomes by differential centrifugation: Theoretical analysis of a commonly used protocol

**DOI:** 10.1038/srep17319

**Published:** 2015-11-30

**Authors:** Mikhail A. Livshts, Elena Khomyakova, Evgeniy G. Evtushenko, Vassili N. Lazarev, Nikolay A. Kulemin, Svetlana E. Semina, Edward V. Generozov, Vadim M. Govorun

**Affiliations:** 1 Engelhardt Institute of Molecular Biology, Russian Academy of Sciences, 32, Vavilova str., Moscow, 119991, Russia; 2 Fededal Research and Clinical Center of Physical-Chemical Medicine, 1a, Malaya Pirogovskaya str, Moscow, 119435, Russia; 3 Moscow Institute of Physics and Technology, 9 Institutskiy Per., Dolgoprudny, 141700, Russia; 4 Faculty of Chemistry, Lomonosov Moscow State University, 1/73, Leninskie gory, Moscow, 119991, Russia; 5 Research Institute of Carcinogenesis, N. N. Blokhin Russian Cancer Research Center, 24 Kashirskoye Shosse, Moscow, 115478, Russia

## Abstract

Exosomes, small (40–100 nm) extracellular membranous vesicles, attract enormous research interest because they are carriers of disease markers and a prospective delivery system for therapeutic agents. Differential centrifugation, the prevalent method of exosome isolation, frequently produces dissimilar and improper results because of the faulty practice of using a common centrifugation protocol with different rotors. Moreover, as recommended by suppliers, adjusting the centrifugation duration according to rotor K-factors does not work for “fixed-angle” rotors. For both types of rotors – “swinging bucket” and “fixed-angle” – we express the theoretically expected proportion of pelleted vesicles of a given size and the “cut-off” size of completely sedimented vesicles as dependent on the centrifugation force and duration and the sedimentation path-lengths. The proper centrifugation conditions can be selected using relatively simple theoretical estimates of the “cut-off” sizes of vesicles. Experimental verification on exosomes isolated from HT29 cell culture supernatant confirmed the main theoretical statements. Measured by the nanoparticle tracking analysis (NTA) technique, the concentration and size distribution of the vesicles after centrifugation agree with those theoretically expected. To simplify this “cut-off”-size-based adjustment of centrifugation protocol for any rotor, we developed a web-calculator.

Differential centrifugation is a widely employed technique for the isolation and purification of different biological objects, such as viruses, cells, subcellular organelles, proteins, and nucleic acids that are dissolved or dispersed in biologically relevant solvents^1^. Centrifugation causes particles that are heavier than the solvent to sediment. The original solution typically contains a multicomponent mixture of particles that differ in sizes and densities and therefore also in their sedimentation rates. The differential centrifugation technique attempts to selectively sediment the components of interest, optimizing the protocol for several rounds of centrifugation.

Differential centrifugation consists of successive centrifugation steps with increasing centrifugation forces and durations, generally aimed at isolating smaller from larger objects. Larger particles, assigned to be removed in the first centrifugation steps, sediment faster and leave most of the smaller particles in the supernatant. The supernatant will be centrifuged in subsequent steps. However, because the initial distribution of all particles within the centrifugation tube is homogeneous, a proportion of the target small particles inevitably co-sediment and may be lost in the withdrawn pellet of large particles. Small particles that start their migration at positions close to the pellet may finish simultaneously with larger particles that traverse longer distances. The differential centrifugation works well only when sedimentation coefficients of the particles to be distinguished differ significantly (by orders of magnitude) and is much less successful when only small differences in sedimentation rates are noted^2^.

A theoretical analysis of differential centrifugation considers the particles as sedimenting independently from each other. Therefore, the theory is basically identical to that developed by the pioneers^3^,^4^ of analytical ultracentrifugation. However, specific problems of preparative centrifugation arise in rather popular modern practices using “fixed-angle” (earlier called “angle-head”) rotors. The early attempts of the theoretical description of this case^5^,^6^ were not successful. Returning to this question, we now suggest an adequate description.

One of the fast-growing fields applying differential centrifugation is extracellular vesicles (EV) research. Cells are known to secrete a number of membranous vesicles differentiated by their sizes, molecular content and by the mechanisms of their formation^7^,^8^,^9^,^10^,^11^,^12^, depending on the type and current state of the cells. Three major populations of EV are usually discerned: apoptotic bodies, shedding vesicles and exosomes^9^,^10^. Apoptotic bodies, the largest of the known vesicles with a diameter of 800–5000 nm, are composed of cytoplasmic and plasma membrane components of post-apoptotic cells. Shedding vesicles and exosomes are released by non-apoptotic cells. Shedding vesicles, also referred to as “ectosomes” or sometimes “microvesicles”, are generated by the blebbing of the plasma membrane and were thought to cover a broad range of sizes (50–1000 nm). However, according to recent observations, the range of their sizes may be much narrower, at least for select systems^13^,^14^. Exosomes are the population of small (40–100 nm) vesicles of endocytic origin. Both exosomes and shedding vesicles contain specific sets of proteins, RNAs and, as described recently^15^, dsDNA and are generally recognized as agents of intercellular communication^16^,^17^. These vesicles are found to occur in cell cultures and in natural body fluids – in blood, saliva, urine and breast milk.

Cells of a single type release both exosomes and shedding vesicles; thus, the extracellular environment is enriched with different types of extracellular vesicles^9^,^10^. Even when formed by the identical cell type, different classes of vesicles are characterized by distinct nucleic acids profiles^18^. However, the exhaustive profiling of exosomes remains a challenge because no reliable approach is available to obtain pure populations of exosomes with a sufficiently high yield.

Exosomes attract enormous research interest because they are promising in important medical applications^19^. Exosomes may serve as diagnostic tools^20^,^21^,^22^ because they are carriers of molecular markers of many diseases^20^,^23^,^24^, including cancer^25^,^26^, and as a prospective delivery system for various therapeutic agents^22^,^27^,^28^,^29^.

The most widely used method for exosome isolation is differential centrifugation^9^,^30^,^31^. The successive rounds of centrifugation are intended to pellet consecutively the apoptotic bodies and cell debris, the shedding vesicles and the exosomes. However, because of the similarity of sedimentation properties of different types of extracellular vesicles, the differential centrifugation applied for exosome isolation often displays unsatisfactory results, providing relatively low yields^32^ and an insufficient purity of the exosome population^33^,^34^,^35^. Moreover, the identical centrifugation protocols are commonly applied with different rotors and without accounting for the differences in viscosity of the solutions^36^, which often leads to a dissimilarity of the data obtained by different research groups. Notably, this heterogeneity is likely responsible for the differences in the reported proportions of exosomes and shedding vesicles.

In this study, we theoretically analyse the sedimentation of vesicles through a differential centrifugation technique using different types of rotors. The theory considers the geometrical parameters of the rotor, the rotation speed, the centrifugation duration and viscosity of the solution and allows the evaluation of the proportion of pelleted vesicles of definite sizes and densities. The “individual” differential centrifugation protocol, adequate for the rotor in use, can be selected by relatively simple theoretical estimates.

## Results

### Theory

The velocity of a particle within the centrifugation tube, as determined by the balance of three forces – centrifugal force, Archimedes floatation force and Stokes viscous drag force – is the following^3^:


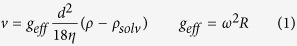


where *g*_*eff*_ is the centrifugal force (acceleration), *R* is the radius of rotation, i.e., the distance of the particle from the rotation axis, ω is the angular frequency of rotation, *d* is the diameter of the particle, *ρ* is the mass density of the particle, and *ρ*_*solv*_ and *η* are the density and viscosity of the medium, respectively. At fixed centrifugation conditions (rotation speed and medium composition) the particle velocity is determined exclusively by the morphology and the density of the particle. A particle with a density higher than the density of the medium moves “down” along the centrifugal force, whereas a particle lighter than the medium “floats up” in the direction opposite to the centrifugal force. For similar densities, larger particles migrate faster. In biology, therefore, centrifugation is mainly applied for the separation of different objects by their size (differential and rate zonal centrifugation) or by their density (isopycnic centrifugation). In this study, we concentrate on particle fractionation by size, using differential centrifugation.

In contrast to isopycnic and rate zonal centrifugation, differential centrifugation starts from a homogeneous initial distribution of particles within the centrifugation tube. A proportion of target small particles situated near the tube bottom inevitably co-sediments with larger particles during the first rounds of centrifugation. This co-sedimentation leads to a decrease in the final yield of smaller particles. However, select large particles, initially localized close to the tube meniscus, may experience an insufficient time to reach the tube bottom during the first centrifugation rounds and thus contaminate the pellet of small particles resulting from the last centrifugation step. Evidently, the degree of cross contamination is dependent on comparative sedimentation velocities of different populations of particles and on the centrifugation conditions. The differential centrifugation protocol can be effectively optimized to obtain both a high yield and sufficient purity of the target particle population in cases of significant (orders of magnitude) differences between the sedimentation velocities of the particles being separated. Additionally, the optimization process is less successful when only small differences are noted in the sedimentation rates between different fractions of particles. In these cases, depending on centrifugation conditions, either the yield of small particles will be low (at higher RPMs and/or longer centrifugation times assuring complete sedimentation of larger particles during the first centrifugation rounds) or the final pellet of small particles will be cross-contaminated with larger particles (at soft conditions in the first centrifugations, i.e., lower RPMs and/or shorter centrifugation times).

Any exosome isolation protocol aims to obtain a population of exosomes with a reasonable yield and without contamination by cell debris, organelles, and apoptotic bodies and, ideally, free from other types of extracellular vesicles, proteins and their aggregates. Separating the exosomes from cells, cell debris and large vesicles is relatively easy because of the large difference in sizes. The substantial challenge is the purification of exosomes from small shedding vesicles because of the close similarity of their sizes. The separation of these extracellular vesicles by differential centrifugation is difficult and inefficient.

The centrifugation protocol should be adapted to consider both the properties of the rotors and characteristics of the particles to be separated.

Because the centrifugal velocity is proportional to the rotation radius of a particle, 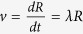, the centrifugal motion is accelerated. The radial coordinate of a particle grows exponentially with time *t*:


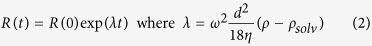


**Figure 1:**
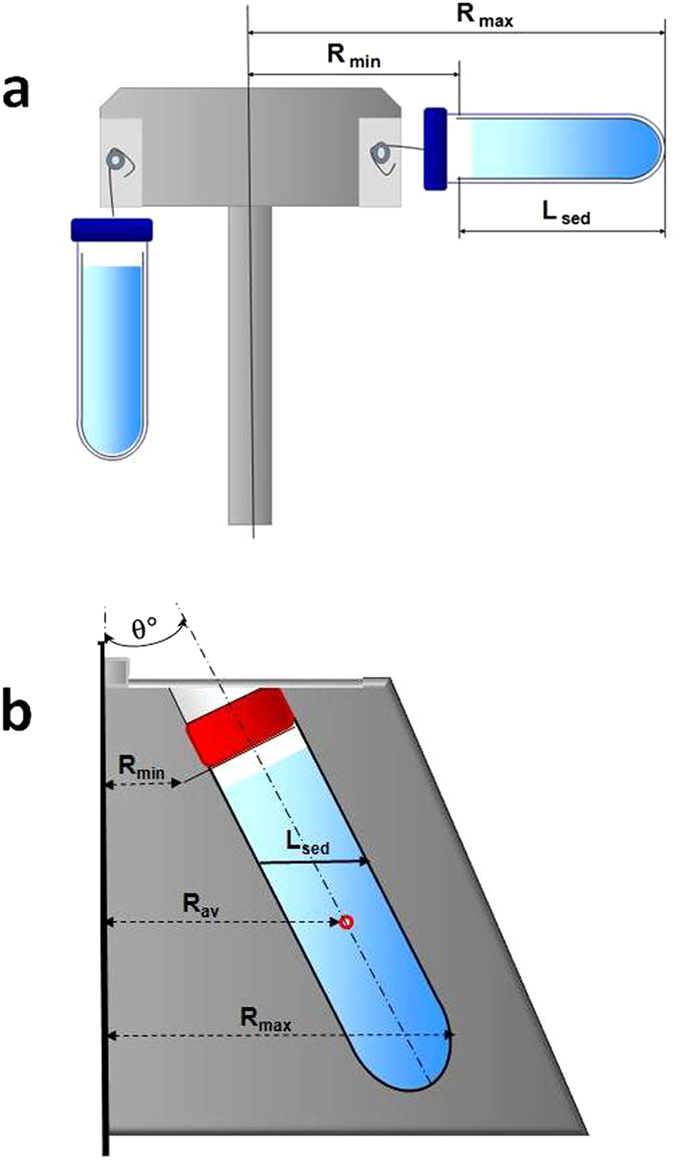
Schematic sketches of the two types of rotors: (**a**) “swinging bucket” and (**b**) “fixed angle”.

Two types of rotors are commonly used in microvesicles research^30^,^35^: “swinging bucket” (SW) rotors (Fig 1A) and “fixed-angle” (FA) rotors (Fig. 1B). These rotors are fundamentally different in their geometry and consequently in their sedimentation properties. The major differences between these rotors that are important for the theoretical description of sedimentation include the following. For FA-rotors, the maximal path length for sedimenting particles usually is small in comparison with the rotation radius, allowing an approximation of the constant migration rate and simplifying the description. However, the second difference is that the horizontal cross-sections of a circular FA-tube are elliptical and the path lengths of different particles differ depending on the distance of the trajectory from the long axis of the ellipse. Peripheral particles may sediment faster because of the shorter path length.

A particle moving by centrifugal force becomes sedimented after reaching the tube bottom in case of a SW-rotor or the tube wall opposite to the rotation axis in case of a FA-rotor. The longest path to run have the particles initially situated at the tube meniscus in case of a SW-rotor and those starting at the tube wall nearest to the rotation axis in case of a FA-rotor (Fig. 1a,b). This distance, *L*_*sed*_, is usually referred to as the *sedimentation path length* of the rotor. The sedimentation path length *L*_*sed*_ in FA-rotors typically is shorter than in SW-rotors. In a FA-rotor, however, the vesicles that reached the tube wall may continue gliding down the tube wall until the final pelleting at the bottom of the tube. The time actually required for vesicles to reach the bottom of the tube cannot be predicted theoretically. The non-compactness of the pellet is one of the main disadvantages of FA rotors, complicating the harvesting procedure and leading to a significant loss of target vesicles. For FA rotors, we select a sedimentation time as the time required for the particles to reach the tube wall opposite to the rotation axis. In the experimental portion of this study, we collect and dissolve the particles from the tube wall and from the tube bottom.

### SW rotor

The complete sedimentation of particles of a given size and density in a SW rotor is achieved when the particles beginning their migration at the position *R*_min_ nearest to the rotation axis have sufficient time to transverse the entire sedimentation path length, *L*_*sed*_ = *R*_max_ − *R*_min_, and reach the pellet at the bottom of the centrifugation tube (*R*_max_, Fig. 1, A). The corresponding conditions (marked by asterisk *) are the following:


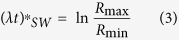


This equation indicates that the centrifugation time required for complete sedimentation of *d*-size particles 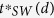 is the following:


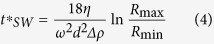


The complete sedimentation time in a SW-rotor, t*_SW_, is dependent on properties of both the rotor and particles and can be determined through the K-factor of the rotor:


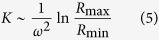


and the sedimentation coefficient ***s*** of the particles:


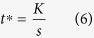


The sedimentation (mobility) coefficient ***s*** is defined by the following equation, correlating velocity with centrifugal force:


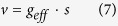


Therefore, according to the equations above, the following can be written:


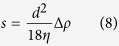


The sedimentation coefficient has the dimensionality of time and the generally accepted unit for its evaluation is Svedberg: 1 S = 10^−13^ sec. With explicit indication of dimensionalities (after the slash) the expressions above take the following form:


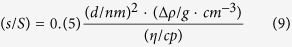



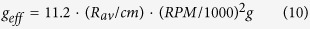


Here, g = 980 cm sec^−2^ is acceleration of gravity.


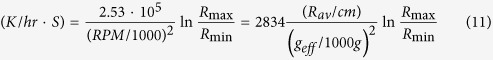


Smaller values of the K-factor require shorter centrifugation times to sediment a given population of particles completely.

Equation (3) determines the cutting-off size d*, i.e., the least size of particles completely sedimented during the time *t* of centrifugation:


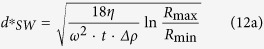


In terms of sedimentation coefficients the “cutting-off” corresponds to:


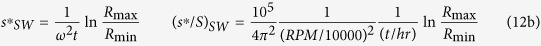


Initially, the particles are homogeneously distributed within the tube along the tube axis from *R*_min_ to *R*_max_. During the time *t* of incomplete sedimentation, the pellet collects only those particles which were initially located within the segment 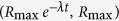 adjacent to the bottom. Consequently, the proportion of pelleted *d*-size particles at centrifugation time *t* < t* (shorter than the time of complete sedimentation of this population) is the following:


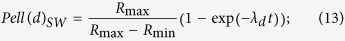


The proportion of pelleted *d*-size particles as dependent on the cut-off size d* represents the losses in target *d*-population because of unavoidable co-sedimentation with the d*-population:


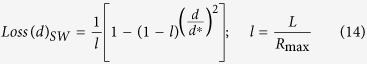


At the limit *l*   1, this equation would produce the following: 
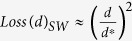


### FA rotor

For FA-rotors, the manufacturer’s documentation usually contains the data on *R*_max_ and *R*_min_. However, using these data in the evaluation of the sedimentation time would be incorrect. The sedimentation path length for FA-rotors is not the difference R_max_ − R_min_, but is determined by the diameter and incline of the tube. Instead, the *R*_max_ and *R*_min_ for a FA-rotor should be *R*_av_ + *L/2* and *R*_av_ – *L/2*, respectively. The complete sedimentation conditions for the particles of a given size and density are the following:


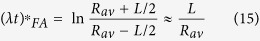


Equation (15) determines both the time required for complete sedimentation of *d*-size particles *t*_FA_*(*d*) and the cutting-off size *d*_FA_*(*t*) for a fixed centrifugation time *t*:


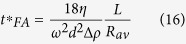



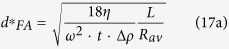


The “cutting-off” sedimentation coefficient is:


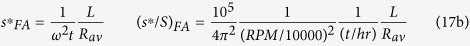


To estimate the proportion of pelleted *d*-size particles for a FA-rotor, the centrifugal migration of particles must be considered within the elliptical horizontal cross-sections of the tube. The path lengths of different particles depend on the distance from the long axis of the ellipse. Peripheral particles sediment faster because of a shorter path length. However, these particles are few in number. To consider the indicated effects, we need to calculate the area of the crescent-shaped segment of the elliptical cross-section, which was originally occupied by the sedimenting particles getting to the pellet during the time *t* of centrifugation. The inner boundary of the segment is formed by the ellipse and is shifted “back” by a distance of *v*_*d*_ *t*. This area, related to the entire area of the ellipse, represents the proportion of sedimented particles. The result of the calculation (see Supplement) is the following:


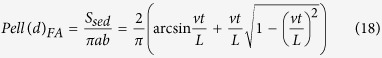



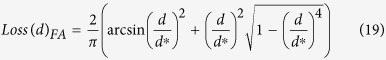


Without considering the elliptical form of the horizontal cross-sections of the FA-tube, which indicates neglecting the presence of shorter peripheral trajectories, a lower sedimentation efficiency would be predicted:


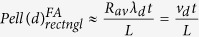


The predictions display the greatest difference at the lowest sedimentation degrees:


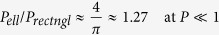


These predictions become identical when approaching the complete sedimentation limit: 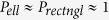.

At levels of semi-complete sedimentation, the difference is on the order of 20%.

The density of exosomes is considered an average value^12^ ρ = 1.15 g∙cm^−3^ (consequently *Δρ* = 0.15 g∙cm^−3^), and the viscosity of the solvent (water)^37^ η(4 °С) = 1.55 cp = 1.55 10^−2^ g cm^−1^ sec^−^^1^. We further develop the expressions for the main observable variables convenient for making numeric estimates.

The centrifugation time required for complete sedimentation of *d*-size particles can be evaluated through the following equations:


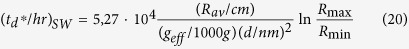



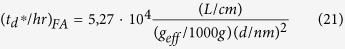


The cut-off size d*, i.e., the least size of particles completely sedimented during the time *t* of centrifugation, is the following:


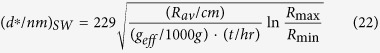



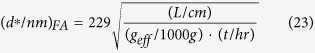


Equations (13,18), representing the time-dependent proportions of pelleted *d*-particles, may be supplemented by the following auxiliary expressions:


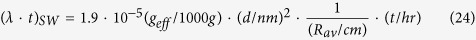



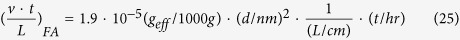


To simplify the analogous calculations of t*, d*, Pell (d), and Loss (d) in cases of particle densities (ρ) different from 1.15 g cm^−3^ and viscosities of the solution (η) different from 1.55 cp = 1.55 10^−2^ g cm^−1^ sec^−1^, we designed an interactive web-calculator available at the following domain: http://vesicles.niifhm.ru/.

### Comparison

We then compare the expected efficiency of the different rotors used for vesicles sedimentation.

Although centrifugation conditions should be adapted to the rotor and to the sizes and densities of the particles to be sedimented, the identical centrifugation protocols are commonly applied on different rotors, leading to significant differences in yield and purity of exosome preparations^35^.

According to the most common protocol^30^, exosome isolation is realized through four consequent centrifugation steps: 10 min at 300 g, 10 min at 2000 g, 30 min at 10000 g, followed by exosome pelleting by centrifugation at 100000 g for 70 min. A repeated 100000 g centrifugation of the re-suspended pellet is usually applied for to purify the exosome preparation from the lower mobility fractions, mainly from free proteins.

The first two rounds of this protocol (low-speed centrifugations at 300 g and 2000 g) are aimed at pelleting the cells, cell debris and large vesicles. The subject of special attention is the 10000 g centrifugation, which (according to the general idea of the method) is intended to pellet the vesicles with diameters exceeding 100–150 nm, presumably corresponding to the population of shedding vesicles. A recent study^38^ showed that a certain amount of exosomes is also pelleted at this step. However, the purification of exosomes from shedding vesicles by centrifugation at 10000 g seems to be incomplete. To our knowledge, no quantitative comparison of the exosomes/shedding vesicles sedimentation efficiency under 10000 g centrifugation in different rotors has been previously performed.

Evidently, the proportions of sedimented exosomes and of shedding vesicles are dependent on the rotor type. We considered the rotors commonly applied for exosome isolation, namely “Fixed Angle” rotors Type 45 Ti, Type 70 Ti, TLA-110 (Beckman) and F-45–24–11 (Eppendorf) and “Swinging Bucket” rotors SW40, SW28 and MLS-50 (Beckman) (Table 1).

**Figure 2:**
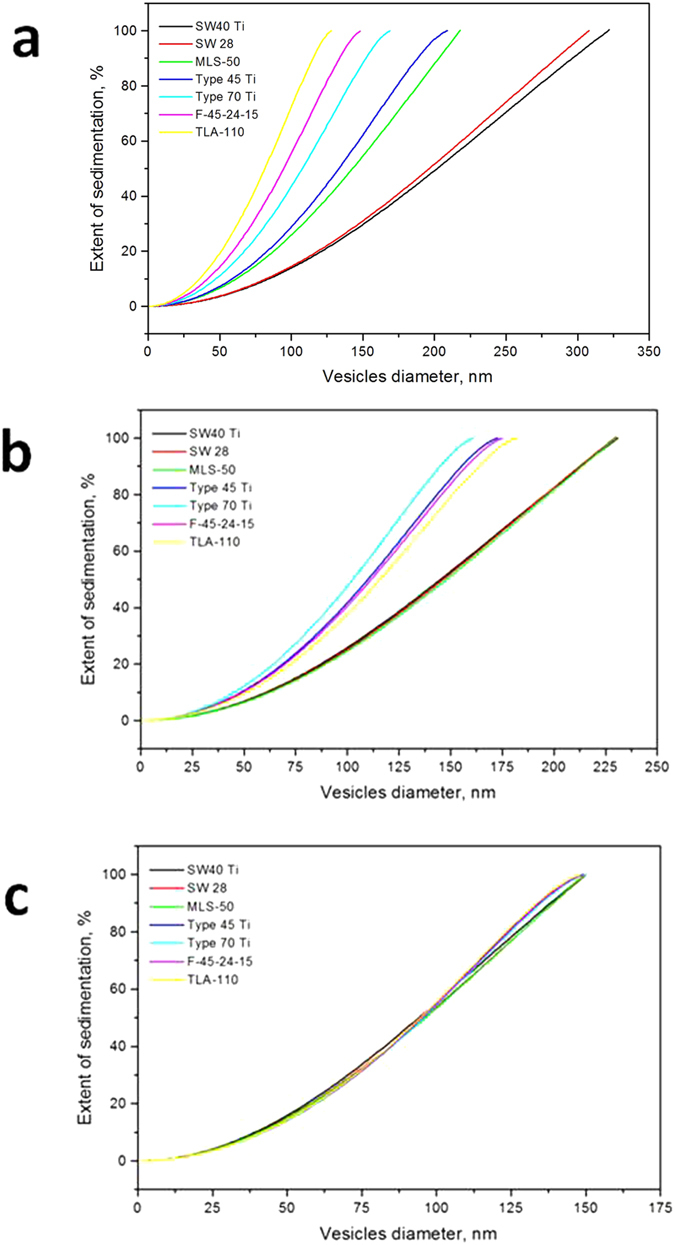
Expected vesicle size-dependent extents of pelleting by centrifugation at RCF = 10000 g with the rotors specified in the insets. (**a**) equal centrifugation time lengths (30 min); (**b**) the time lengths are adjusted according to the “K-factor rule”; (**c**) time lengths of the centrifugation correspond to a definite size (150 nm) of complete sedimentation (“cut-off-size” rule).

Figure 2a shows the expected proportions of sedimented vesicles of various sizes after 30 min of centrifugation at 10000 g, calculated according to equations (13, 24, 18, 25) for the rotors, listed above. Table 2 represents the corresponding “cut-off” sizes of vesicles d*(30 min, 10000 g) and the proportions of pelleted 150 nm, 120 nm, 100 nm and 70 nm vesicles. The application of the identical protocol for various rotors should result in drastically different size-dependences of pelleting.

A commonly accepted recipe for equalizing the sedimentation efficiency of different rotors is the adjustment of the centrifugation times proportionally to K-factors of the rotors (see eqs. (5, 11)). However, because the K-factor is well defined exclusively for SW rotors, this approach is inadmissible for FA rotors. Nevertheless, we produce estimates based on K-factors for both SW and FA rotors to obtain an authentic view of the scale of the inconsistencies. Characterizing the MLS-50 swinging bucket rotor (as a reference) by K-factor K_MLS_, the centrifugation in another (“new”) rotor should be considered equivalent when the centrifugation durations are proportional to the K-factors:


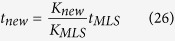


The K-factors and adjusted centrifugation times *t*_K_, represented in Table 3 were calculated according to equation (11) for SW and FA rotors (although improperly for FA-rotors). Using the K-factor values, the adjusted centrifugation times were estimated according to equation (26) and applied in equations (13, 24, 18, 25) to evaluate the “cut-off” sizes and pelleting levels for vesicles of smaller sizes (Table 3 and Fig. 2b). The calculations demonstrate that the adjustment of the centrifugation durations using the K-factor does perfectly equalize the centrifugation efficiency in the case of SW-rotors, but not in the case of FA-rotors. The expected sedimentation profiles for FA rotors remain different after the K-factor based “equalization”.

The preferential method to adjust the centrifugation protocol to a specific rotor consists of selecting the proper centrifugation duration t* for the definite cut-off size of sedimented particles d* on the basis of equations (4, 16, 20, 21). The estimates represented in Table 4 and in Fig. 2c, corresponding to cutting off size d* = 150 nm, demonstrate a remarkable equalization of the expected co-sedimentation losses of smaller particles, even between SW- and FA-rotors. This close similarity of the losses in SW- and FA-rotors (which are described by differing eqs. (14, 19)), theoretically is not indispensable.

When calculating the 10000 g sedimentation profiles, represented above in Fig. 2a,b,c and Tables 2,4, we used the commonly accepted mean value 1.15 g/cm^3^ for the density of the vesicles. However, because of the uncertainty in the experimental determination of the density, we also estimate the expected results of the final sedimentation at 100000 g centrifugation for different densities in the range from 1.08 to 1.19 g/cm^3^. Table 5 demonstrates the dependence of the expected cut-off-sizes of the completely sedimented vesicles on their density for a 70 min centrifugation at 100000 g d*(70 min, 100000 g) in various rotors. For the mean density value of 1.15 g/cm^3^, the 70 min 100000 g centrifugation in FA-rotors seems to be sufficient for pelleting the entire population of exosomes. However, when the real density of exosomes is lower, e.g., 1.12 or even 1.08 g/cm^3^, a considerable proportion of smaller exosomes may avoid pelleting and be lost in the supernatant. Moreover, a repeated centrifugation of re-suspended pellet will additionally increase the losses. For SW-rotors with long path-lengths, the 70 min centrifugation at 100000 g hardly can be regarded as sufficient for the sedimentation of the entire exosome population. The centrifugation time necessary for the complete sedimentation of vesicles larger than approximately 40 nm, t*(40 nm), is easily evaluated using the d*(70 min) values of Table 5: 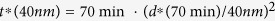. At a vesicle density value as low as 1.08 g/cm^3^, the rotor-dependent sedimentation time t*(40 nm) varies from approximately 6 hours for the SW40 Ti rotor to less than 1 hour for the TLA110 (FA type) rotor. Conversely, at presumably the highest vesicle density of 1.19 g/cm^3^, the exosome sedimentation time t*(40 nm) varies between approximately 2.5 hours and 23 min for the above-mentioned rotors.

### Experiment

As an illustration of the appropriateness of our theoretical consideration, we present experimental data for exosomes isolated from HT29 cell culture using a standard purification protocol^30^. The pellet of the last step of the preparation (100000 g centrifugation) was re-suspended in PBS buffer in a volume equal to the initial volume of the supernatant to give the starting exosome population for subsequent test centrifugations at 10000 g. This step of differential centrifugation (10000 g) is expected to be sensitive to the selection of the rotor (see Fig. 2). For the test, we use pre-purified exosomes to minimize the influence of secondary details such as viscosity. The concentration and particle size distribution (PSD) of the purified exosomes were determined by the Nanoparticle Tracking Analysis (NTA) technique.

**Figure 3:**
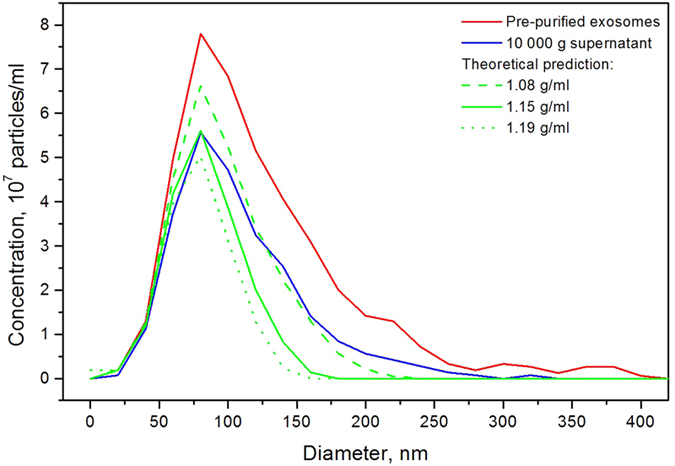
The size distribution of the HT29 exosome population changing under a test centrifugation (30 min, 10000 g) in a Beckman 60 Ti rotor: (red)–NTA distribution of pre-purified exosomes before the test centrifugation; (blue)–measured NTA distribution observed after the centrifugation; (green)–theoretically expected distributions for the assumed vesicle densities: (solid) 1.15 g/cm^3^, (dashed) 1.08 g/cm^3^, (dotted) 1.19 g/cm^3^.

We compare the results of the test centrifugation with those theoretically expected. A FA-type rotor (Beckman Type 60 Ti), being of special interest in the test because of the novelty of the theoretical description, was considered (Fig. 3). NTA PSD of the supernatant after 30 min centrifugation at 10000 g was compared with the theoretically expected distribution, calculated as superposition of the initial NTA PSD of pre-purified exosomes and the theoretically predicted proportion of vesicles of each size, which is to remain in supernatant after the testing centrifugation *1-Pell(d)*_*FA*_. The calculation is performed with the assumption of the generally accepted mean vesicle density value (1.15 g/cm^3^) and for lower (1.08 g/cm^3^) and higher (1.19 g/cm^3^) values.

Figure 3 shows the compared NTA distributions of the initial pre-purified exosomes (red curve) and supernatant after 30 min centrifugation at 10000 g in a Type 60Ti rotor (blue curve). Green curves represent the theoretically expected distributions for vesicles of different densities. Figure 3 displays a good accordance between the theoretical and measured NTA PSDs for the subpopulation of small (30–100 nm) vesicles, confirming the predictions for the exosome-like vesicles with an average density of 1.15 g/cm^3^. The excess of measured larger vesicles (over 100 nm) could be explained by two factors. First, NTA distributions are artificially widened because of finite particle track lengths. Second, when the density of larger vesicles is lower than that of exosomes (1.08 g/cm^3^), these lower-density large vesicles sediment slower and may cause an excess of larger vesicles observed in the 10000 g supernatant. Overall, the correspondence of calculated and observed vesicles size distributions confirms our theoretical predictions for fixed-angle rotors.

**Figure 4:**
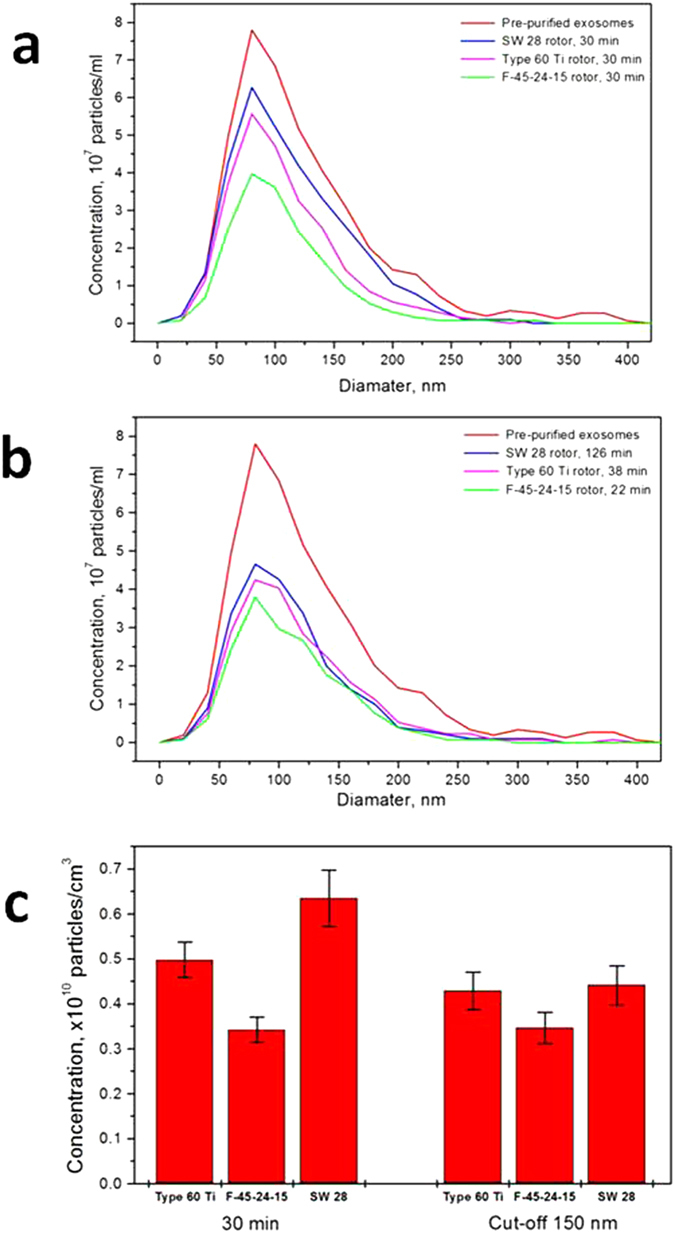
Comparison of the “general protocol” centrifugation strategy with that using a “cut-off-size” based adjustment of the centrifugation duration for three different rotors. **(a)** the NTA size distributions changed because of the 30 min centrifugation at 10000 g in the three rotors: (Beckman Type 60 Ti, Eppendorf F-45–24–15 and Beckman SW28); (**b**) the NTA size distributions after centrifugation with the duration adjusted to the complete sedimentation of 150 nm vesicles and larger; (**c**) the histograms of the total vesicle concentrations.

Using three different rotors – two FA-type rotors (Beckman Type 60 Ti and Eppendorf F-45–24–15) and one SW-type rotor (Beckman SW28), we compare the experimental results of the traditional “general protocol” strategy and those obtained with the suggested “cut-off-size”-based adjustment of centrifugation duration. Figure 4a shows the NTA PSD of the initial pre-purified exosomes (red curve) and the PSDs obtained after 30 min of 10000 g centrifugation in SW28, Beckman Type 60 Ti and Eppendorf F-45–24–15 rotors (blue, magenta and green curves correspondingly). The distributions differ substantially. Therefore, different vesicle populations are obtained by equally long centrifugations in different rotors. By contrast, Fig. 4b shows that when the centrifugation durations for different rotors are selected as those corresponding to the complete sedimentation of vesicles of a definite size (“cut-off-size”), the differences in the NTA distributions of vesicle populations in different rotors diminish. Figure 4c displays the histograms of the vesicle concentrations and demonstrates the efficiency of the “cut-off-size”-based “equalization” of the sedimentation abilities of various rotors.

## Discussion

The present work aimed to detail the theoretical analysis of vesicle isolation by differential centrifugation using various rotors.

We adapted the general equation describing the velocity of a particle sedimenting under centrifugation force to a form that would be convenient for calculating the sedimentation profile. The proportion of pelleted vesicles of a given size as dependent on the centrifugation force, duration and sedimentation path length for the specific rotors in use may be estimated by anyone. The theoretical analysis clearly shows that the recommendations of commonly used differential centrifugation protocol in terms of centrifugation force g_eff_ and duration accepted without accounting for specific parameters of the rotors may be misleading. All performance criteria, such as the yield and the purity of target population of vesicles and the required duration of centrifugations, were appreciably dependent on the type of the rotors (“swinging bucket” or “fixed angle”) and on their specific parameters (rotation radius, sedimentation path length). We demonstrate that the rather common practice of a K-factor-based adjustment of the centrifugation duration at a change of the centrifuge is unjustified and erroneous for “fixed-angle” rotors. We propose another method to equalize the sedimentation efficiency of centrifugation with different rotors – the “cut-off-size”-based method. The centrifugation duration required for complete sedimentation of the vesicles of a given size (and larger) calculated for the specific rotor and the selected rotation speed (or for a given centrifugal force) may serve as the basis for the development of the individual differential centrifugation protocol adequate for the centrifuge available. Additionally, the results of the isolation and purification of exosome preparations obtained in different laboratories are comparable when their centrifugation protocols are correlated through “cut-off-size”-based equalization. Main theoretical statements were confirmed by test experiments on exosomes isolated from HT29 cell cultures.

The theory and the recommendation of the “cut-off” - based protocols of differential centrifugation may be helpful in many other applications of preparative centrifugation aimed at isolation of various biological samples including viruses, proteins, ribonucleoprotein complexes and others.

## Methods

### Cells

The HT29 human colorectal adenocarcinoma cell line (ATCC HTB-38) was cultured in 225 cm^2^ cell culture flasks in DMEM medium (Sigma, USA) supplemented with 10% FBS (Gibco, USA), 2 mM glutamine, penicillin/streptomycin at 37 °C and 5% CO_2_. At 80–90% confluence, the cells were washed three times with PBS, and the medium was changed to 20 ml serum-free DMEM.

### Isolation of exosomes from HT29-conditioned medium by differential centrifugation

Cell culture supernatant (160 ml) was harvested at 24 h and centrifuged at 300 g (1290 rpm) for 10 minutes at 4 °C to pellet dead cells and bulky debris (Eppendorf Centrifuge 5804 R, A-4–44 swinging-bucket rotor) followed by centrifugation at 2000 g_av_ (3333 rpm) for 10 min at 4 °C in the identical rotor to eliminate debris and large vesicles. The supernatant was then centrifuged at 10000 g for 30 min in a Type 60 Ti fixed angle rotor at 4 °C. After centrifugation, the supernatant was collected and subjected to 100000 g centrifugation in a Type 60 Ti rotor (38000 rpm) for 70 min at 4 °C. The 100000 pellet was suspended in PBS in a volume equal to the initial volume of supernatant and subjected to test centrifugations at 10000 g in three different rotors: Type 60 Ti, F-45–24–15 and SW28. The final supernatant was collected and subjected to a NTA.

### NTA particle size and concentration measurements

Measurements of particle size distribution (PSD) and concentration were performed with a Nanosight LM10 HS-BF instrument (Nanosight Ltd, UK) based on a Nanoparticle Tracking Analysis (NTA). Measurements were performed with a 405-nm 65-mW laser and an EMCCD Andor Luca camera. Samples were diluted with particle-free PBS (pH = 7.4) to reach the optimal concentration for NTA according to ASTM E2834–12^39^. All measurements were performed under the identical camera settings (Shutter: 850, Gain: 450, Lower Threshold: 910, Higher Threshold: 11180, 60 s) and processing conditions (NTA 2.3 build 0033, Detection Threshold: 9 Multi, min Track Length: Auto, min Expected Size: 30 nm). Measurements were performed in several repeats (15–25) to collect at least 5000 particles. To obtain the joint histogram of the PSD for multiple measurements, single particle diameters and track lengths from each measurement were collected into a global table and binned with weights proportional to track lengths. A decrease of the total vesicle concentration during the storage (at 4 °C) was considered.

## Additional Information

**How to cite this article**: Livshts, M. A. *et al.* Isolation of exosomes by differential centrifugation: Theoretical analysis of a commonly used protocol. *Sci. Rep.* **5**, 17319; doi: 10.1038/srep17319 (2015).

## Supplementary Material

Supplementary Information

## Figures and Tables

**Table 1:** Specification of typical rotors.

Rotor type	Rotor name	R_min_, mm	R_max_, mm	R_av_, mm	D, mm	Angle, °	L_sed_, mm
SW	SW 40Ti	66.7	158.8	112.8	–	–	92.1
	SW28	75.3	161	118.2	–	–	85.7
	MLS-50	47.5	95.8	71.7	–	–	48.3
FA	Type 45 Ti	35.9	103.8	69.9	38	24	41.6
	Type 60 Ti	36.9	89.9	63.4	25	23.5	27.3
	Type 70 Ti	39.5	91.9	65.7	25	23	27.2
	F-45–24–15	54	82	68	11	45	15.6
	TLA 110	26	48.5	37.3	13	28	14.7

**Table 2:** Expected results of a 30 min centrifugation at RCF = 10000 g with the rotors specified in the rows. “Cut-off” sizes of completely sedimented vesicles d* (nm) and proportions of pelleted 150 nm, 120 nm, 100 nm and 70 nm vesicles are evaluated.

Rotor type	Rotor name	d* (30 min)	Pell (150 nm)	Pell (120 nm)	Pell (100 nm)	Pell (70 nm)
SW	SW 40Ti	321 nm	30%	20%	14%	7%
	SW28	308 nm	31%	21%	15%	7%
	MLS-50	230 nm	51%	34%	25%	12%
FA	Type 45 Ti	210 nm	62%	41%	29%	14%
	Type 60 Ti	170 nm	88%	61%	43%	22%
	Type 70 Ti	169 nm	88%	62%	43%	22%
	F-45–24–15	128 nm	100%	95%	73%	38%
	TLA 110	125 nm	100%	98%	76%	40%

**Table 3:** Evaluation of the results of the K-factor-based adjustment of centrifugation times *t*_*K*_, min for the rotors specified in the rows.

Rotor type	Rotor name	K-factor	t_K_ (according to K-factor)	d*(t_K_)	Pell (150 nm)	Pell (120 nm)	Pell (100 nm)	Pell (70 nm)
SW	SW 40 Ti	**2774.6**	58 min	231 nm	53%	36%	26%	13%
	SW 28	**2547.2**	54 min	229 nm	52%	35%	25%	13%
	MLS-50	**1426**	30 min	230 nm	51%	34%	25%	12%
FA	Type45Ti	**2103.9**	44 min	173 nm	86%	59%	42%	21%
	Type60Ti	**1601**	34 min	159 nm	96%	68%	49%	24%
	Type70Ti	**1573.8**	33 min	161 nm	94%	67%	48%	24%
	F-45–24–15	**765.9**	16 min	175 nm	84%	57%	41%	20%
	TLA 110	**658.9**	14 min	182 nm	79%	53%	38%	19%

RCF = 10000 g. Expected “cut-off”-sizes of completely sedimented vesicles d*(*t*_K_) (nm) and pelleting levels (%) for vesicle sizes 150 nm, 120 nm, 100 nm and 70 nm are presented in the corresponding columns.

**Table 4:** Expected results of the “cut-off-size”-based adjustment of centrifugation times for different rotors.

Rotor type	Rotor name	t* (150 nm)	Pell (120 nm)	Pell (100 nm)	Pell (70 nm)
SW	SW 40 Ti	138 min	74%	55%	30%
	SW 28	126 min	72%	54%	29%
	MLS-50	71 min	72%	53%	28%
FA	Type 45 Ti	59 min	76%	55%	28%
	Type 60 Ti	38 min	75%	54%	27%
	Type 70 Ti	38 min	75%	54%	27%
	F-45–24–15	22 min	76%	55%	28%
	TLA 110	21 min	76%	55%	28%

The pelleting levels for vesicle sizes 120 nm, 100 nm and 70 nm are evaluated for a centrifugation at RCF = 10000 g during the time *t* *(150 nm), ensuring the complete sedimentation of 150 nm vesicles.

**Table 5:** Vesicle density dependence of the expected cut-off-sizes d* of completely sedimented vesicles for a 70 min centrifugation at 100000 g.

Vesicles density	1.08 g/cm^3^	1.12 g/cm^3^	1.15 g/cm^3^	1.17 g/cm^3^	1.19 g/cm^3^
SW 40 Ti	91 nm	74 nm	66 nm	62 nm	59 nm
SW 28	87 nm	71 nm	64 nm	60 nm	57 nm
MLS-50	65 nm	53 nm	48 nm	45 nm	42 nm
Type 45 Ti	59 nm	48 nm	43 nm	41 nm	39 nm
Type 60 Ti	48 nm	39 nm	35 nm	33 nm	31 nm
Type 70 Ti	48 nm	39 nm	35 nm	33 nm	31 nm
TLA 110	35 nm	29 nm	26 nm	24 nm	23 nm
