# Corrigendum: Isolation of exosomes by differential centrifugation: Theoretical analysis of a commonly used protocol

**DOI:** 10.1038/srep21447

**Published:** 2016-03-29

**Authors:** Mikhail A. Livshits, Elena Khomyakova, Evgeniy G. Evtushenko, Vassili N. Lazarev, Nikolay A. Kulemin, Svetlana E. Semina, Edward V. Generozov, Vadim M. Govorun

Scientific Reports 5: Article number: 1731910.1038/srep17319; published online: 11302015; updated: 03292016

The original version of this Article contained a typographical error in the spelling of the author Mikhail A. Livshits, which was incorrectly given as Mikhail A. Livshts. This has now been corrected in both the PDF and HTML versions of the Article.

