# Supplementary material for: Isolation of exosomes by differential centrifugation: Theoretical analysis of a commonly used protocol
Source: Sci Rep. 2015 Nov 30;5:17319. doi: 10.1038/srep17319 (PMC4663484; doi:10.1038/srep17319)
Supplement: Supplementary Information [file srep17319-s1.pdf]

“Isolation of exosomes by differential centrifugation: Theoretical analysis of a commonly used protocol”
by
Mikhail A. Livshts, Elena Khomyakova, Evgeniy G. Evtushenko, Vassili N. Lazarev, Nikolay A. Kulemin, Svetlana E. Semina , Edward V. Generozov, Vadim M. Govorun

Supplement

The estimate of the pelleting efficiency of the FA-rotor when accounting for the differences in sedimentation path lengths within elliptical horizontal cross-sections of the tube.

The centrifugation path lengths for FA-rotors typically are rather small in comparison with the rotation radius. This small length allows the velocities of migrating particles to be considered constant during centrifugation. Particles of the identical size and the identical density have the identical velocity ***v***. In a horizontal elliptical cross-section of the tube, the particles move in the direction parallel to the long axis of the ellipse. Those particles, which arrive at the boundary (wall of the tube) opposite to the rotation axis, become sedimented. During the time ***t*** of centrifugation this occurs with the particles that were initially situated in the crescent-shaped area between the “finishing ellipse” and the ellipse imaginarily shifted “back” by a distance of ***vt*** (see **Figure S1**.). The magnitude of the crescent-shaped area S_{sed} related to the entire area of the ellipse $S_{ell} = \pi ab$ (*a* and *b* are the long and short half-axes of the ellipse, respectively) characterizes the sedimentation efficiency (the proportion of pelleted particles).

$$S_{sed} = 2b \int_{-vt/2}^{a} dx \sqrt{1 - x/a^{\,2}} - 2b \int_{vt/2}^{a} dx \sqrt{1 - x/a^{\,2}} = 2b \int_{-vt/2}^{vt/2} dx \sqrt{1 - x/a^{\,2}}$$

After the substitution $x = a\sin\varphi, \quad y = b\cos\varphi, \quad \frac{vt}{2} = a\sin\Phi$, the following equation is obtained:

$$S_{sed} = 2ab \int_{-\Phi}^{\Phi} \cos^2\varphi d\varphi = 2ab \;\; \Phi + \sin\Phi\cos\Phi$$

Finally, the proportion of pelleted particles is the following:

$$P_{sed} = \frac{S_{sed}}{S_{ell}} = \frac{2}{\pi} \;\; \Phi + \sin\Phi\cos\Phi \;\; = \frac{2}{\pi}\left(\arcsin\frac{vt}{2a} + \frac{vt}{2a}\sqrt{1 - \left(\frac{vt}{2a}\right)^2} \right)$$

More precise estimates of the pelleting efficiency are required for FA-rotors with path lengths comparable to the rotation radii. Considering the velocity growth because of the centrifugal displacement of particles, the inner boundary of the crescent shaped area and the “starting contour” $x_s(y)$ are not an exact copy of the “finishing ellipse” $x_f(y)$, but is related to $x_f(y)$ by the following mapping:

$$x_s + R \;\; e^{\lambda t} = x_f + R$$

The X-coordinate of the cusp (– c) is determined by the following relationship:

$$-c = x_s \;\; x_f = c \;\; = ce^{-\lambda t} - R \;\; 1 - e^{-\lambda t} \qquad\qquad c = R\frac{1-e^{-\lambda t}}{1+e^{-\lambda t}}$$

The area of interest as the integral over the long axis coordinate ξ is the following:

$$S_{sed} = 2\int d\xi \left[y_f \;\; \xi \;\; - y_s \;\; \xi \;\; \right]$$

The Y-coordinate on the ellipse is readily found from the ellipse equation is the following:

$$y_f \;\; \xi \;\; = b\sqrt{1-\frac{\xi^2}{a^2}}$$

The Y-coordinate on the prototype is determined through the mapping above:

$$y_s \;\; \xi \;\; = b\sqrt{1-\frac{x_f \;\; \xi \;\;{}^2}{a^2}}\text{, here } x_f \;\; \xi \;\; = \xi e^{\lambda t} + R \;\; e^{\lambda t} - 1 \;\; = \alpha\xi + \beta, \quad \alpha = e^{\lambda t},$$

$$\beta = R \;\; e^{\lambda t} - 1$$

$$S_{sed} = 2b\int_{-c}^{a_s} d\xi \left[\sqrt{1-\frac{\xi^2}{a^2}} - \sqrt{1-\frac{\alpha\xi+\beta \;\;{}^2}{a^2}} \right] + 2b\int_{a_s}^{a} d\xi \sqrt{1-\frac{\xi^2}{a^2}} \qquad a_s = ae^{-\lambda t} - R \;\; 1 - e^{-\lambda t}$$

$$S_{sed} = 2b\int_{-c}^{a} d\xi \sqrt{1-\frac{\xi^2}{a^2}} - 2be^{-\kappa t}\int_{c}^{a} d\xi' \sqrt{1-\frac{\xi'^2}{a^2}}$$

$$\int_{\pm c}^{a} d\xi \sqrt{1-\frac{\xi^2}{a^2}} = \frac{a}{2}\left[\frac{\pi}{2} \mp \varphi_c \mp \frac{c}{a}\sqrt{1-\left(\frac{c}{a}\right)^2} \right] \qquad \sin\varphi_c = \frac{c}{a} = \frac{R}{a}\frac{1-e^{-\lambda t}}{1+e^{-\lambda t}}$$

The proportion of sedimented particles in the elliptical cross-section is the following:

$$P_{sed}^{ell} = \left[\frac{1-e^{-\lambda t}}{2} + \frac{\varphi_c}{\pi} \;\; 1+e^{-\lambda t} \;\; + \frac{c}{\pi a}\sqrt{1-\left(\frac{c}{a}\right)^2} \;\; 1+e^{-\lambda t} \;\; \right]$$

The complete sedimentation corresponds to the following: $\lambda t^* = \ln\frac{R+a}{R-a}$

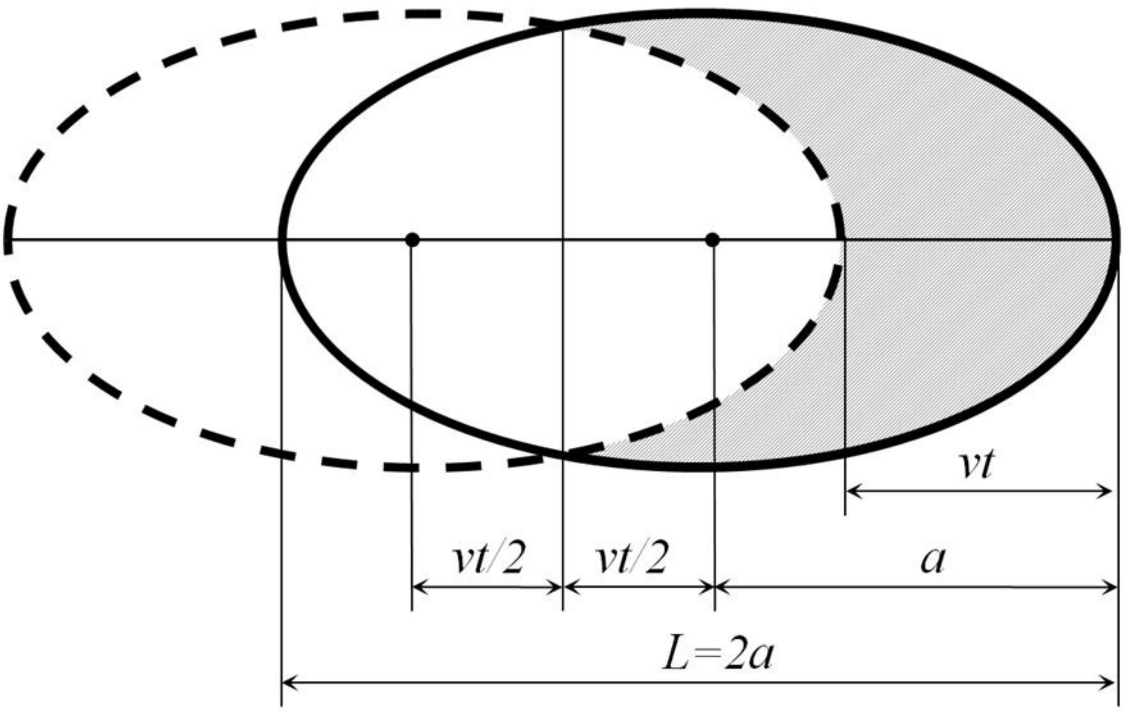

Figure S. An elliptic horizontal cross-section of FA-tube is shown. Of all particles, moving uniformly within the ellipse, only those arrive at the boundary during the time ***t*** of centrifugation, which were initially situated not further than ***vt*** from the boundary, i.e. in the hutched area.
